# A Guideline for the Management of Gastrointestinal Stromal Tumour (GIST)

**DOI:** 10.1080/1357714021000045217

**Published:** 2002-09

**Authors:** Ian Judson, Michael Leahy, Jeremy Whelan, Paul Lorigan, Mark Verrill, Robert Grimer, Martin Robinson

**Affiliations:** 1 Royal Marsden Hospital London UK; 2 St James's Hospital Leeds UK; 3 Middlesex Hospital London UK; 4 Christie Hospital Manchester UK; 5 Newcastle General Hospital Newcastle UK; 6 Royal Orthopaedic Hospital Birmingham UK; 7 Weston Park Hospital Sheffield UK; 8 FRCP Cancer Research UK Centre for Cancer Therapeutics Institute of Cancer Research 15 Cotswold Rd, Sutton Surrey SM2 5NG UK

## Abstract

Gastrointestinal stromal tumour (GIST) is the commonest mesenchymal tumour to affect the gastrointestinal tract. Appropriate management requires accurate diagnosis and the skills of a multidisciplinary team. Surgery is the only curative treatment option and should be performed whenever feasible, by experienced personnel. For patients with advanced unresectable or metastatic disease, the receptor tyrosine kinase inhibitor imatinib offers effective therapy and can provide effective palliation for the majority of patients with this disease. The background to this recent development and a guideline for the management of GIST is proposed.


*Sarcoma* (2002) **6,** 83–87

Taylor & Francis healthsciences

**ORIGINAL ARTICLE**

### A guideline for the management of gastrointestinal stromal tumour (GIST)

IAN JUDSON[1], MICHAEL LEAHY[2], JEREMY WHELAN[3], PAUL LORIGAN[4], MARK VERRILL[5], ROBERT GRIMER[6] & MARTIN ROBINSON[7]

[1]*Royal Marsden Hospital, London, UK,* [2]*St James's Hospital, Leeds, UK,* [3]*Middlesex Hospital, London, UK,* [4]*Christie Hospital, Manchester, UK,* [5]*Newcastle General Hospital, Newcastle, UK,* [6]*Royal Orthopaedic Hospital, Birmingham,* [7]*Weston Park Hospital, Sheffield, UK*

**Abstract**
Gastrointestinal stromal tumour (GIST) is the commonest mesenchymal tumour to affect the gastrointestinal tract. Appropriate management requires accurate diagnosis and the skills of a multidisciplinary team. Surgery is the only curative treatment option and should be performed whenever feasible, by experienced personnel. For patients with advanced unresectable or metastatic disease, the receptor tyrosine kinase inhibitor imatinib offers effective therapy and can provide effective palliation for the majority of patients with this disease. The background to this recent development and a guideline for the management of GIST is proposed.

### Introduction

In early 2002 a group of UK investigators, either members of the European Organisation and Treatment of Cancer (EORTC) Soft Tissue and Bone Sarcoma Group (STBSG) and/or the National Cancer Research Institute Sarcoma Group, UK (current Chair IJ), with experience in the use of imatinib for the treatment of gastrointestinal stromal tumours (GIST) agreed to develop guidelines for the management of GIST, including how to incorporate the use of this drug into UK clinical practice. A licence was granted this year for the treatment of patients with advanced, unresectable or metastatic GIST, on the basis of the data from the Phase II study conducted in the USA. Imatinib is an expensive agent but, owing to its high level of activity in this setting, there is considerable pressure on physicians and health commissioners to make the drug available. This guideline is the result of extensive discussion and consultation with medical oncologists, clinical oncologists, surgeons, and pathologists involved in the management of sarcoma. We have tried to set out recommendations that should help limit inappropriate use of the drug, while at the same time recognising its undoubted utility for patients with advanced GIST.

Gastro-intestinal stromal tumours are mesenchymal cancers (sarcomas) that arise from the connective tissues of the gastro-intestinal tract. Although rare, they are nevertheless the commonest sarcomas to arise in the gut. The incidence of GIST is uncertain but appears to be in the region of 10 per million per year, i.e. 500–1000 patients a year in the UK. These tumours were previously classified as leiomyosarcoma, but in recent years it has become accepted that they are a distinct entity and can usually be distinguished from leiomyosarcoma by expression of the cell surface markers CD34 and CD117. These markers are shared with the interstitial cells of Cajal, or pacemaker cells and it has been suggested that GISTs arise from Cajal cells or a common progenitor cell.[1,2] Cajal cells form a network in the myenteric plexus of the gut and have a role in the coordination of peristalsis.

GISTs commonly have gain of function mutations in the KIT gene that codes for the KIT receptor tyrosine kinase. This is the receptor for stem cell factor and is designated CD117 in terms of immunohistochemistry. The commonest exon 11 mutations allow receptor dimerisation in the absence of stem cell factor, making the signalling pathway independent of external influence.[3] GISTs have different degrees of aggressiveness, resulting in variation in their potential, and time taken, to metastasise.

Correspondence to: Prof Ian Judson MD, FRCP, Cancer Research UK Centre for Cancer Therapeutics, Institute of Cancer Research, 15 Cotswold Rd, Sutton, Surrey SM2 5NG, UK. Tel: +44-20-8722-4302; Fax: +44-20-8642-7979; E-mail: judson@icr.ac.uk

ISSN 1357–714X print/ISSN 1369–1643 online/02/0200083–05 © Taylor & Francis Ltd
DOI: 10.1080/1357714021000045217

GISTs occur predominantly in middle aged and older people with about 70% arising in the stomach. Patients with surgically resectable GISTs have a 10-year survival rate of 30–50%. The median survival of patients with surgically unresectable, metastatic and advanced sarcomas of soft tissue is about 12 months.[4] Experience has shown that these tumours are even less responsive to chemotherapy than other sarcomas, with a response rate estimated at <5%,[5] in other words the standard chemotherapy agents used for other sarcomas, such as doxorubicin and ifosfamide, have no place in the management of the disease.

### Imatinib

Imatinib (Glivec, STI 571) was granted a licence in the EU for the treatment of advanced unresectable or metastatic GIST in May 2002. A dose of 400 or 600 mg daily is recommended, based on data from the US Phase II trial, which explored these doses. It is acknowledged that the optimum dose may vary from patient to patient. Imatinib is an orally administered, selective inhibitor of the BCR-ABL, KIT, PDGF-R and ARG tyrosine kinases. Phase I, II and III trials have consistently shown that the majority of patients with locally advanced unresectable or metastatic disease benefit from treatment with imatinib. Imatinib is generally given at a starting dose of 400 mg/day orally for chronic myeloid leukaemia (CML), for which it was licensed in November 2001. NICE recently published a final appraisal determination on the use of imatinib in chronic myeloid leukaemia (http://www.nice.org.uk) taking into account results from a trial indicating a high incidence of complete cytogenetic response, 85% progression-free and 91% overall survival at 24 months in chronic phase patients receiving imatinib. The official guidance on which groups of patients are to be recommended treatment with imatinib will be published shortly. The issue of the most appropriate dose for GIST is discussed below but there are some data indicating that larger doses might be necessary for optimal treatment of GIST compared with CML.

### *Research data in support of the use of imatinib in GIST*

Imatinib was developed as an inibitor of BCR-ABL for the treatment of chronic myeloid leukaemia and has proved highly effective against the disease. It was recognised that functional expression of the receptor tyrosine kinase KIT in GIST, often due to activating mutations in KIT, might make imatinib a useful drug in this setting. A single patient with GIST was treated successfully in the Spring of 2000, clinical trials began in the USA in July and in Europe in August 2000. A Phase II study in the USA compared 400 with 600 mg daily. Those progressing on 400 mg/day were transferred to 600 mg. Thirty-five patients were evaluable. Partial responses, assessed at 1–3 months, were seen in 19 patients (54%), stable disease in 12 (34%). Four patients (11%) progressed. Median survival had not yet been reached (patient recruitment from July to September 2000) at the time of study report.[6]

A Phase I study in Europe studied doses of 400, 600, 800 or 1000 mg daily, in 40 patients with advanced soft tissue sarcomas (36 of whom had GISTs). Preliminary data were reported in November 2001.[7] Tumour inhibition was seen in 30 patients with GIST, with 19 confirmed partial responses and six unconfirmed partial responses. A recent update demonstrated that 29 (80%) of the patients with GIST were still on treatment after a minimum of 11 month.[8] Doses of 1000 mg/day were likely to cause dose-limiting side effects, but 800 mg daily was feasible. The commonest side effects were skin rash, nausea and vomiting, oedema and fatigue. Some myelosuppression was seen but this was rarely dose-limiting. FDG-PET scan responses a week after the start of treatment predicted subsequent computed tomography (CT) responses.

A Phase II study was performed at 800 mg daily in patients with GIST and other soft tissue sarcomas, and was reported at the recent American Society of Clinical Oncoloy meeting.[9] A response rate of 71% (one complete and 18 partial remissions) was demonstrated in the GIST patients, whereas no objective remissions were observed in other sarcomas. Most notable was the fact that only 11% of patients with GIST progressed during the first 8 weeks of therapy, whereas the progression rate in other sarcomas was 71%. Although it has been noted that GIST tumours may become less radiodense on CT scan at an early stage, objective responses may be slow to materialise, sometime occurring many months later, with a median time to remission of 4 months. Serious side effects were rare and grade 2 toxicities other than anaemia, oedema and fatigue occurred in less than 10% of patients. Most toxicities tended to diminish in severity with time. With complete data available at a minimum of 11 months of follow-up, 73% of patients were free from progression at 12 months. Overall survival at 12 months was >95%. Phase III trials comparing 400 and 800 mg daily have been performed in the USA, coordinated by the National Cancer Institute, and in Europe, conducted by the European Organisation for Research and Treatment of Cancer, both of which have now closed. To date only safety data are available, indicating that imatinib is an extremely safe medication.[10,11] A similar level of clinical benefit in the Phase III trial has been observed by individual investigators, to that reported in the original Phase I and Phase II trials.

It is clear from the limited data available that median survival for patients requiring treatment has increased from under a year to well over a year. In the original Phase I trial, the study that currently has the longest follow-up, the updated information presented at the American Society of Clinical Oncology meeting (Orlando, FL, May 2002), 27 of 35 (77%) patients with GIST were still receiving treatment at 16 months minimum follow-up. These patients were either in partial remission or had stable disease, four of them had required a dose escalation in order to regain control of their disease. Five had relapsed and died after an initial response or disease stabilisation. Symptomatic benefit in that study was 80%.[7]

It must be emphasised that these figures fail to do justice to the dramatic improvements that have been seen. Imatinib clearly causes rapid tumour cell kill in many patients, who experience rapid relief of pain, abdominal distension and anorexia. Weight gain can be dramatic and some patients have been rescued from terminal cancer cachexia, at a stage in their disease when they had already lost more than 25% of their body weight, with serum albumin values of significantly less than 30 g/l. In this situation, survival can usually be measured in weeks but many of these patients remain alive and well, often in full time employment, a year or more after starting treatment.

Additional studies are required to advance our understanding of what constitutes the optimum dose for treatment of advanced disease and the role of KIT mutations in determining response or resistance. However, it was reported that two patients progressing on 400 mg had disease stabilisation on 800 mg daily,[11] and patients relapsing after an initial good response may also sometimes be stabilised on a higher dose (van Oosterom, ASCO, 2002). The updated response rate of 71% in the 800-mg daily Phase II study might also indicate the presence of a dose–response relationship, given that this is somewhat higher than that seen in the Phase I trial over the whole dose range. However, these are non-randomised data and we must await mature response data from the Phase III trial. Tumour shrinkage may occur very slowly. Data presented recently[8] indicate a median of 4 months from start of treatment to objective remission, with some patients taking much longer. Often the first objective evidence of response is clinical softening of tumour masses and radiological 'liquefaction', i.e., tumours becoming cystic.

It is known that certain KIT mutations (exon 11) are favourable for response to imatinib while patients with the wild-type gene are much less likely to respond. A recent paper showed that KIT activation, as indicated by a characteristic gene expression pattern, can occur in the absence of KIT immunohistochemical staining.[12] Perhaps in the future it will be appropriate to carry out mutational analysis prior to initiating treatment. Similarly, it appears that that the likelihood of response can be predicted with some accuracy using an early PET scan.

**Recommedations**

See algorithm in Fig 1.

- The diagnosis of GIST needs to be made by suitably experienced pathologists. The choice of antibody for assessment of CD117 expression appears to be important and use of the DAKO antibody has been recommended in all large trials performed to date, preferably without antibody retrieval techniques, which can result in false-positives. It must be emphasised that the diagnosis of GIST does not rest on CD117 expression alone. It should be made by pathologists with experience of the disease, and needs to take into account morphology and other immunohistochemical markers, such as CD34. In addition, albeit rarely, CD117 negative GIST has been described. Recent data indicate that such tumours may still have activating KIT mutations.
- Like other sarcomas, GISTs should be managed in an appropriate multidisciplinary setting, ideally a Multidisciplinary Sarcoma Team. The principal treatment for GIST is surgery, which may be curative, but patients should be discussed pre-operatively and some might benefit from pre-operative treatment if operability is borderline. It is likely that 4–6 months' of treatment would be required in order to obtain maximum benefit from pre-operative therapy. Operations should be performed by surgeons with experience in the management of sarcomas.
- Imatinib should be considered the treatment of choice for patients with advanced unresectable or metastatic GIST. It would be appropriate for healthcare commissioning bodies to designate centres for the treatment of GIST with imatinib in order to ensure that adequate experience is gained with this expensive drug in such a rare tumour type. The drug has significant toxicity and requires careful monitoring. There is still much to be learnt about its optimal delivery and it is anticipated that further trials will be conducted to explore the biology of the disease in relation to its response to imatinib, and to define the role of adjuvant and pre-operative therapy.
- Patients with advanced disease should be treated before they develop severe cachexia. As with other anticancer agents, the incidence of side effects correlates with declining performance status and earlier treatment is likely to be both better tolerated and more effective. It may also delay the emergence of drug resistance.
- Currently the recommendation would be to initiate treatment with imatinib given at a dose of 400 mg daily P.O. with food. Higher doses may be given in the event of a poor response or relapse after initial response, but should not be continued beyond 8 weeks in the absence of clear cut clinical and radiological benefit. These recommendations may be modified in the light of mature results from the Phase III trials.

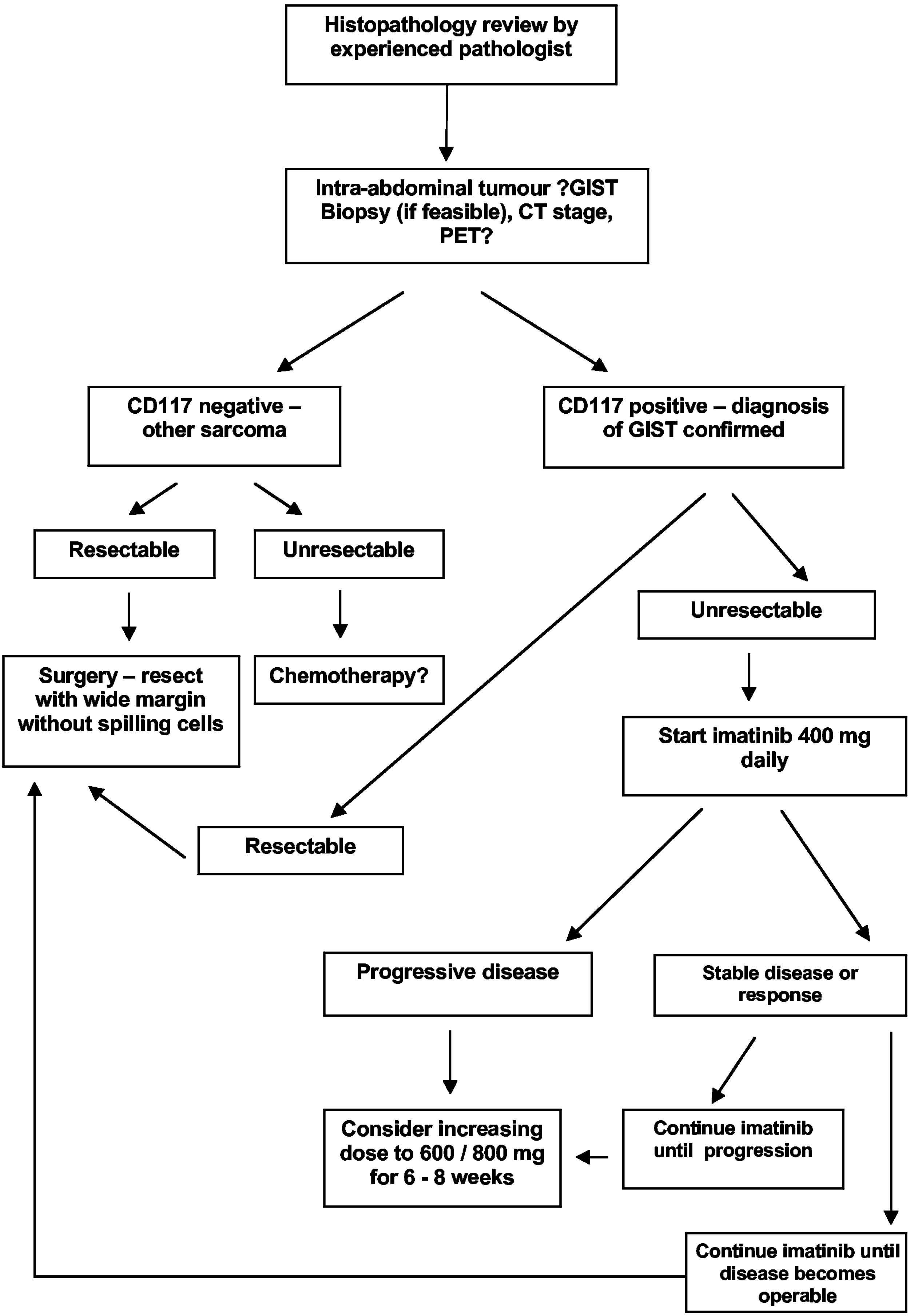

**Fig. 1.** *Algorithm for management of GIST.*

### References

1 Kindblom L-G, Remotti HE, Aldenborg F, *et al.* Gastrointestinal pacemaker cell tumour (GIPACT): gastrointestinal stromal tumours show phenotypic characteristics of the interstitial cells of Cajal. *Am J Pathol* 1998; 152: 1259–69.

2 Sircar K, Hewlett BR, Huizinga JD, *et al.* Interstitial cells of Cajal as precursors of gastrointestinal stromal tumours. *Am J Surg Pathol* 1999; 23: 377–89.

3 Hirota S, Isozaki K, Moriyama Y, *et al.* Gain-of-function mutations of c-kit in human gastrointestinal stromal tumors. *Science* 1998; 279; 577–80.

4 Van Glabbeke M, van Oosterom AT, Oosterhuis JW, *et al.* Prognostic factors for the outcome of chemotherapy in advanced soft tissue sarcomas: an analysis of 2,185 patients treated with anthracycline-containing first-line regimens – a European Organisation for Research and Treatment of Cancer Soft Tissue and Bone Sarcoma Group Study. *J Clin Oncol* 1999; 17: 150–7.

5 Platt BE, Hollema H, Molenaar WM, *et al.* Soft tissue leiomyosarcomas and malignant gastrointestinal stromal tumours: differences in clinical outcome and expression of multidrug resistance proteins. *J Clin Oncol* 2000; 18: 3211–20.

6 Blanke CD, von Mehren M, Joensuu H, *et al.* Evaluation of the safety and efficacy of an oral molecularly-targeted therapy, STI571, in patients with unresectable or metastastic gastrointestinal stromal tumours (GISTs) expressing C-KIT (CD117). *Am Soc Clin Oncol* 2001; 20; Abstr 1a.

7 Van Oosterom AT, Judson I, Verweij J, *et al.* for the European Organisation for Research into Treatment for Cancer (EORTC), Safety and efficacy of imatinib (STI571) in metastatic gastrointestinal stromal tumours; a phase I study. *Lancet* 2001; 358: 1421–3.

8 Van Oosterom AT, Judson I, Verweij J, *et al.* Update of the imatinib (STI571, Glivec) phase I study in gastrointestinal stromal tumours (GISTs) *Proc Am Soc Clin Oncol* 2002; Abstr 327.

9 Judson IR, Verweij J, van Oosterom A, *et al.* Imatinib (Gleevec) an active agent for gastrointestinal stromal tumors (GIST), but not for other soft tissue sarcoma (STS) subtypes not characterized for KIT and PDGF-R expression results of EORTC Phase II studies. *Proc Am Soc Clin Oncol* 2002; Abstr 1609.

10 Demetri G, Rankin C, Fletcher C, *et al.* Phase III randomised study of imatinib mesylate (Gleevec, STI571) for GIST: intergroup SOO33 early results. *Proc Am Soc Clin Oncol* 2002; Abstr 1651

11 Casali PG, Verweij J, Zalcberg J, *et al.* Imatinib (Glivec) 400 mg vs 800 mg daily in patients with gastrointestinal stromal tumours (GIST) a randomised phase III trial from the EORTC Soft Tissue and Bone Sarcoma Group, the Italian Sarcoma Group (ISG) and the Australasian Gatro-Intestinal Trials Group (AGITG). A toxicity report. *Proc Am Soc Clin Oncol* 2002; Abstr 1650.

12 Nielsen TO, West RB, Linn AC, *et al.* Molecular characterisation of soft tissue tumours: a gene expression study. *Lancet* 2002; 359: 1301–7.
